# Extraction and characterization of phenolic compounds and their potential antioxidant activities

**DOI:** 10.1007/s11356-022-23337-6

**Published:** 2022-10-06

**Authors:** Linghong Shi, Wanrong Zhao, Zihong Yang, Vigasini Subbiah, Hafiz Ansar Rasul Suleria

**Affiliations:** School of Agriculture and Food, Faculty of Veterinary and Agricultural Sciences, The University of Melbourne, Parkville, VIC 3010 Australia

**Keywords:** Plant, Phenolic compound, Extraction, Separation, Characterization, Antioxidant capacity

## Abstract

For thousands of years, plant has been widely applied in the medical area and is an important part of human diet. A high content of nutrients could be found in all kinds of plants, and the most outstanding group of nutrients that attracts scientists’ attention is the high level of phenolic compounds. Due to the relationship between high phenolic compound content and high antioxidant capacity, plant extracts are expected to become a potential treatment for oxidation stress diseases including diabetes and cancer. However, according to the instability of phenolic compounds to light and oxygen, there are certain difficulties in the extraction of such compounds. But after many years of development, the extraction technology of phenolic compounds has been quite stable, and the only problem is how to obtain high-quality extracts with high efficiency. To further enhance the value of plant extracts, concentration and separation methods are often applied, and when detailed analysis is required, characterization methods including HPLC and LC/GC–MS will be applied to evaluate the number and type of phenolic compounds. A series of antioxidant assays are widely performed in numerous studies to test the antioxidant capacity of the plant extracts, which is also an important basis for evaluating value of extracts. This paper intends to provide a view of a variety of methods used in plants’ phenolic compound extraction, separation, and characterization. Furthermore, this review presents the advantages and disadvantages of techniques involved in phenolic compound research and provides selected representative bibliographic examples.

## Background

In 2600 BC, people in Mesopotamia developed around 100 plant-derived substances for medical purposes. Around 5000 years ago, natural drug molecules were recorded in the Indian Ayurveda system’s record. And in Chinese literature, countless use cases of herbs are recorded since 2500 years ago (Alves and Rosa 2007; Ekor 2014). But not just in the medical area, plants are also an important part of human’s daily diet. The human regular dietary sources can be divided into two parts, animal foods and plant foods. As Chen et al. (2018) mentioned in their work, plant foods have unique nutrients that do not exist in animal foods, so they can complement people’s daily dietary intake. In addition to the common nutrients such as minerals and protein that also exist in animal foods, the high nutrient value of plants also comes from their unique dietary fiber and phenolic compounds.

The content of phenolic compounds is one of the most important factors that evaluate the health effects of plant foods. Antimutagenic, antidiabetic, and anti-inflammatory functions of plant products were highly related to the antioxidant capacity brought by the content of phenolic acids (Biglari et al. 2008; Vayalil 2002). Other polyphenols like procyanidins and flavonoids were also found in plants (Habib et al. 2014; Mansouri et al. 2005). Nonetheless, as Hong et al. (2006) mentioned in their work, due to different stages of maturity, the presence and concentration of some of the glycosylated flavonoids and procyanidins may also be different. Therefore, the measurement of phenolic compounds in plants is necessary to make the best use of plants.

## Phenolic compounds in plants

Four main branches of polyphenols can be found in plant foods, which can be classified as flavonoids, stilbene, lignin, and phenolic acids based on their structure differences, phenol ring numbers, and molecular linkage type (Manach et al. 2004). Polyphenols provide antioxidant capacity which has many health effects such as assisting the human body to achieve better glycemic control, hypotensive, and increasing lipid profile (Li et al. 2015; Rodrigo et al. 2008; Zemestani et al. 2016). The antioxidant capacity of polyphenols is also expressed in the form of inhibiting the oxidative process by scavenging free radicals and reactive oxygen species which can help prevent oxidative stress diseases including cancer and diabetes (Ademiluyi and Oboh 2013; He and Sun 2016).

Phenolic acid is one of the main types of phenolic compounds present in plants. Scientists have already found a high concentration of phenolic acids in plants’ seeds, skins, and leaves which are present in bound form. Usual form of phenolic acids is found in amides, esters, and glycosides forms but seldom in free form (Pereira et al. 2009). According to the research done by Clifford (1999), hydroxybenzoic acid and hydroxycinnamic acid are the two sub-groups of phenolic acids. Hydroxycinnamic acids are often present in food as simple esters with quinic acid and glucose. Hydroxybenzoic acids are derived from benzoic acid and are found in soluble form and bound with cell wall fractions such as lignin (Khoddami et al. 2013; Strack and Dey 1997).

Flavonoid is a kind of secondary metabolite that is also rich in plants and contributes to the color, fragrance, and flavor characteristics of plants (Maleki et al. 2019). Due to the presence of hydroxy groups in flavonoids’ chemical structure, the bioavailability and biological activity in human body can be affected. The basic chemical structure of flavonoids is two benzene rings (A and B) linked by a three-carbon pyran ring (C). Due to the position of the B-ring and the position and number of hydroxy groups on it, the antioxidant capacity of flavonoids can be different (D’Amelia et al. 2018). The mechanism of the chemical reaction of flavonoid antioxidant protection is achieved by the donated electrons through resonance from functional hydroxy groups to stabilize free radicals (Šamec et al. 2021). Flavonoids have been widely applied in food and pharmaceutical industries due to their outstanding antioxidant capacity (Kumar and Pandey 2013). However, the industrial use of flavonoids and other phenolic compounds requires high-quality extractions. Therefore, the extraction methods have been developed to increase the production and more in line with the requirement of environmentally friendly (Rodríguez De Luna et al. 2020).

Previous studies have reported that the antioxidant capacities of plant seeds and peel are higher than pulp which is because most phenolics and flavonoids are stored in plant seeds and peel (Alharbi et al. 2021). But the seeds and peel are often discarded during product processing which seems to be a waste of high content of antioxidant compounds. Therefore, to effectively use plant wastes, efficient methods should be applied to extract the high content of phenolic compounds from food wastes.

## Extraction methods

Phenolic compounds exist in plants in the form of glycosides or aglycones, and due to the stability differences, they can also exist as matrix and free-bound compounds. The structure differences can also affect the existence of phenolic compounds making them exist in the form of polymerized or monomers. Phenolic compounds are not universally distributed in plants with different stability which makes the extraction of these compounds more challenging (Alara et al. 2021). Single-step extraction and unsuitable extraction methods may influence the recovery rate of phenolic compounds from plant samples. Therefore, suitable extraction method selection is of vital importance for recovering the target phenolic compounds. To have a deeper understanding of extraction methods for obtaining phenolic compounds from plants, this review will focus on extraction methods that have been widely used which are listed in Table 1.

**Table 1:** The mechanism, advantages, disadvantages, and example(s) of extraction methods

Method	Mechanism	Advantages	Disadvantages	Example(s)
Conventional extraction	The law of similarity and inter-miscibility	Simple in principle and easy to operate	Time consuming and large volume of solvent required	Saafi et al. (2009)
Ultrasonic-assisted extraction (UAE)	Implosion of cavitation bubbles when ultrasonic is applied causing surface peeling, erosion, and particle breakdown to release more phenolic compounds	Short operation time, low in cost, small volume of solvent required	Heat generated during extraction may cause phenolic compound decomposition	Chemat et al. (2017); Wei et al. (2010)
Reflux extraction	Reflux extraction extracts and concentrates the solvent simultaneously. During the extraction process, the solvent in the extraction tank is pumped out into the concentration tank. In the concentration tank, the solvent will be heated until evaporated then condensed and pumped back into the extraction tank. After repeating this process, the extracts in solvent will be accumulated	Short operation time, small volume of solvent required, lower fixed investment	Heating during evaporation process may cause phenolic compound decomposition	Habchi et al. (2021); Kongkiatpaiboon and Gritsanapan (2013); Sultana et al. (2009)
Microwave-assisted extraction (MAE)	Using microwave energy to heat solvents containing samples, thereby partitioning phenolic compounds from a sample matrix into the solvent	Short operation time, lower requirement for space, time, and solvent	High energy cost, the heat generated during applying microwave may cause phenolic compound decomposition, require extra consideration of the sample size	Chen et al. (2007); Hao et al. (2002); Hemwimon et al. (2007); Pan et al. (2002)
Soxhlet extraction	Soxhlet extraction is a modified analytical extraction method based on reflux extraction using a specific extractor called the Soxhlet extractor, which can be used for distillation purposes	Short operation time, small volume of solvent required, easy to operate, suitable for initial and bulk extraction	Heating during evaporation process may cause phenolic compound decomposition	Alara et al. (2018); Aspé and Fernández (2011); Ouahida et al. (2016)
Pressurized liquid extraction (PLE)	Solid sample is packed into a steeled container with extraction solvents then extracted for 5 to 15 min under high temperature and pressure	Shorter operation time, small volume of solvent required, good repeatability	Heating during extraction process may cause phenolic compound decomposition	Ju and Howard (2003); Alonso-Salces et al. (2001)
Supercritical fluid extraction (SFE)	Applying supercritical fluid like supercritical carbon dioxide as its solvent for phenolic compound extraction	Low operation temperature, high selectivity, inertness nontoxic	High requirement for co-solvent selection in phenolic compound extraction; high cost	Vatai et al. (2009); Ashfaq et al. (2021); Vuong et al. (2010)
Pulsed electric field extraction (PEF)	Applying short, high voltage pulses to destroy membrane structures and release more phenolic compounds into solvents	Short operation time, low operation temperature	The membrane changes are reversible, air bubbles make the process less effective, and the efficiency of the method depends on electric field strength and electrode gap	Liu et al. (2018); Joannes et al. (2015)
Enzyme-assisted extraction (EAE)	Enzymes like cellulase can decompose cell membranes which are mainly formed by macromolecules polysaccharides and proteins to increase the content of phenolic compounds released to solvents	Low operation temperature	Long operation time, hard to control, impurities may be also released during extraction	Hai et al. (2016)

### Conventional extraction

Maceration is a conventional extraction that has been widely applied to extract phenolic compounds from plants. Conventional extraction is a technique that uses the law of similarity and inter-miscibility (like dissolves like) to transfer target chemicals into the solvent (Zhang et al. 2018). In maceration extraction, ethanol or methanol is often selected as a solvent for phytochemical investigation which could extract the organic phenolic compounds from the plant samples. To accelerate the extraction progress, a shaking incubator is often used for shaking and temperature control which can increase the extraction rate by increasing the contact surface between samples and solvent. As Zhang et al. (2018) mentioned in their work, higher temperature can increase solubility and diffusion but avoid overheating is also important because if the extraction temperature is too high, the solvent will be lost and phenolic compounds will decompose. A successful example of applying conventional extraction was done by Saafi et al. (2009) using 50% methanol as a solvent to extract phenolic compounds from plants under room temperature. Their extraction process was assisted with an orbital shaker at a frequency of 200 rpm. As a result, the samples they tested were found rich in total phenolic, ranging from 209.42 to 447.73 mg equivalent gallic acid⁄100 g (GAE/100 g) fresh weight.

The extraction efficiency of conventional extraction depends on the extraction duration in a certain time range. Higher extraction duration brings higher efficiency but when a solute equilibrium is achieved between inside and outside solid matter, increasing time will not affect the extraction. To obtain a higher extraction yield, a greater solvent-to-solid ratio is required but when the solvent-to-solid ratio is too high, excessive extraction solvent will be used and requires a long time for extraction. As a result, conventional extraction methods usually use organic solvent maceration which requires a large volume of solvents and long extraction time which is stable but not efficient enough. Granulometry is another important factor that affects extraction efficiency. According to the research done by Mukhopadhyay et al. (2006), the total phenolic contents of black cohosh with four particle sizes (2 mm, 0.85 mm, 0.425 mm, and 0.250 mm) were measured, and the results showed the highest values were obtained from black cohosh with particle size range from 0.25 to 0.425 mm. The reason for this phenomenon is the surface area per unit mass of the plant increases while the particle size decreases which allows more phenolics to release.

### Ultrasonic-assisted extraction (UAE)

To shorten the time required for extraction and increase the extraction quality, ultrasound is used in the solvent-producing cavitation which can bring high shear forces. Micro-jetting will be caused by the implosion of cavitation bubbles when ultrasonic is applied and will cause surface peeling, erosion, and particle breakdown, as do macro-turbulences and micro mixing (Chemat et al. 2017). As a result, it accelerates the dissolution and diffusion of the solute as well as the heat transfer. Compared with conventional extraction, UAE requires less solvent but a shorter extraction time.

Although, stronger ultrasonic applied to materials will create a greater shear force which can accelerate the changes. But due to cost considerations, ultrasonic applications in food industries are usually optimized to achieve the best result with the lowest energy usage (Bermúdez-Aguirre et al. 2011). To achieve the highest efficiency of extraction, increasing ultrasound power, reducing the moisture content in food materials, and temperature control are often considered. And the frequency of ultrasound must be selected properly because it can affect the size of bubbles produced during resonance.

Commonly, the frequencies applied in UAE processes range from 20 to 100 kHz. And for phenolic compounds, Chukwumah et al. (2009) reported changing the frequency can achieve the effect of molecule selection, for example, daidzein and genistein can be extracted at 25 kHz and biochanin A and trans-resveratrol can be extracted at 80 kHz. For regular extraction, 40 kHz was the most suitable frequency. González-Centeno et al. (2014) used the response surface methodology for the study of influencing parameters; the authors tested three frequencies (40 kHz, 80 kHz, and 120 kHz) for grape pomace phenolic compound extraction and found 40 kHz was the most effective. This was also approved by the study done by Almusallam et al. (2021); the optimum UAE condition was found under frequency 40 kHz, temperature 40.8 °C, duration 21.6 min, and ethanol concentration 50.0% which could extract the highest total phenolic content of 130.2 mg GAE from date palm spikelets.

### Reflux extraction

Reflux extraction, also called solvent recycling reflux extraction, is an extraction method that extracts and concentrates the solvent simultaneously (Chen et al. 2013). Reflux extraction is divided into two parts, extraction tank and concentration tank. During the extraction process, the solvent in the extraction tank is pumped out into the concentration tank. In the concentration tank, solvent will be heated until evaporated then condensed and pumped back into the extraction tank. After repeating this process, the extracts in the solvent will be accumulated. Reflux extraction compared with conventional extraction has many advantages including shorter extraction time due to higher mass transfer driving force, less solvent cost due to solvent reuse, and lower fixed investment due to no requirement of a storage tank before concentration (Zhao et al. 2011). However, reflux extraction also has its disadvantages. Due to the requirement of concentration in reflux extraction and heating, phenolic compounds may be contaminated during evaporation or decompose at high temperatures.

Reflux extraction compared with conventional extraction has some advantages but it cannot be a substitute for conventional extraction. Habchi et al. (2021) studied phenolic compound extraction using the reflux method, and the total phenolic content was found to vary from 8.04 and 46.81 mg GAE/100 g dry weight. According to the research done by Kongkiatpaiboon and Gritsanapan (2013), in *Stemona collinsiae* Craib roots extraction, refluxing with 70% ethanol was the most recommended method which could obtain the highest yield of didehydrostemofoline. Also, an investigation of comparing conventional and reflux extraction methods showed no matter what solvent was used for extraction, reflux extracts had a higher extraction rate. But contrary to the extraction rate, higher total phenolic content and antioxidant capacity were obtained by conventional extraction (Sultana et al. 2009).

### Microwave-assisted extraction (MAE)

MAE is an extraction method using microwave energy to heat solvents containing samples, thereby partitioning phenolic compounds from a sample matrix into the solvent. Compared with conventional extraction, selective migration of target compounds could be transferred from materials to surroundings within a shorter time by highly localized temperature and pressure generated during MAE. In addition, the recoveries of MAE are similar or even higher than conventional extraction while the space requirement, time requirement, and solvent requirement are all lower. The achievements of applying MAE in plant phenolic compound extractions have been reported by researchers (Chen et al. 2007; Hao et al. 2002; Hemwimon et al. 2007; Pan et al. 2002).

Similar to conventional extraction, in MAE, only the microwave heating effect can increase the product recovery (Spigno and De Faveri 2009). Although microwave heating is faster than conventional heating which saves time, the energy cost during this process may be higher. Therefore, the comparison of UAE and conventional extraction should bring the punctual energy cost into consideration as electricity is one of the most expensive energy forms. However, if the penetration depth characteristic for the solvent is larger than the sample size, MAE will lead to a dramatic increase in the heating rate that causes super boiling (Spigno and De Faveri 2009). Therefore, when transforming the laboratory scale results of MAE into industrial scale, the solvent and sample size need to be carefully considered to avoid false results and safety problems.

### Soxhlet extraction

Compared with other conventional methods that appear in phenolic compound extraction, Soxhlet extraction requires less solvent and time while the processing cost is also very low. In addition, the extraction device is easy to operate and is suitable for initial and bulk extraction with a good recovery rate (Seidel 2012). Soxhlet extraction is an improved method based on reflux extraction which also integrates the advantage of percolation. Due to reflux and siphon, the fresh solvent can be recycled to extract materials continuously. Soxhlet extraction is automatic, and compared with conventional extraction, the solvent and time required for Soxhlet extraction are less. But Soxhlet extraction is a thermal extraction that may cause thermal degradation during a long extraction time when heating.

Successful examples of phenolic compounds extraction with the Soxhlet method were done by Alara et al. (2018), the highest yields of Soxhlet extraction from *Vitis cinerea* (Engelm.) Engelm. ex Millardet. leaves were obtained using 60% ethanol. Soxhlet extraction as Aspé and Fernández (2011) mentioned in their study had the highest extraction rate compared with MAE, UAE, and conventional extraction. But compared with UAE and MAE, the time required for Soxhlet to achieve the same extraction quality is longer. Soxhlet extraction can obtain a higher total phenolic and tannin content than conventional extraction but the following example showed that if treated with more stages, the phenolic compounds may decompose due to the high temperature. Ouahida et al. (2016) showed that after 6 h of extraction in 70% acetone, 528.81 mg GAE/g of total phenolic content was observed from date leaves. The result was higher than conventional extraction (484.44 mg GAE/g) but lower than UAE (625.17 mg GAE/g).

### Pressurized liquid extraction (PLE)

Common processing of PLE is handled by packing solid samples into a steeled container and filled with extraction solvents then extracted for 5 to 15 min under high temperature and pressure. Due to high pressure, solvents can remain in liquid form at a temperature above their boiling point during PLE which makes the lipid solutes in the solvent in a high solubility and diffusion rate and allows easier penetration of the solvent into the matrix. Compared with conventional extraction, PLE also can decrease the requirement of solvent and time and have better repeatability.

PLE has been widely applied by many scientists to extract natural products like anthocyanin and saponins (Ju and Howard 2003; Zhang et al. 2018). PLE compared with conventional techniques requires same or even lower volume of solvents, and PLE is time-saving but does not need much handling of the sample (Alonso-Salces et al. 2001). Katsinas et al. (2021) mentioned in their work, that by comparing the extraction effect of conventional extraction and PLE, they found that compared with conventional extraction, PLE reduced by 67% extraction time and 38% solvent while the phenolic production increased.

However, as mentioned above, the mechanism of PLE is allowing solvents to remain in liquid form at high pressure and temperature to enhance the extraction rate but this may cause heat degradation. Therefore, Ju and Howard (2003) recommended extraction solvents that are not so efficient at low temperatures like water can be combined with PLE to increase its efficiency.

### Supercritical fluid extraction (SFE)

SFE is an extraction method that applies supercritical fluid (SF) as its solvent for extraction. SF is an ideal solvent that has a similar solubility to liquid while the diffusivity is similar to gas making it able to dissolve a wide range of natural materials. However, SF is sensitive; the solvating properties of SF may change a lot near their critical points caused by small pressure and temperature changes. One of the typical SF used in extraction is supercritical carbon dioxide (S-CO_2_) which has been widely used. The advantages of using S-CO_2_ include low critical temperature (31 °C), selectivity, inertness, and nontoxic can extract compounds that are liable under high temperatures. S-CO_2_ is an ideal solvent for non-polar materials extraction like lipids due to its low polarity and can be enhanced its solvating properties by adding a modifier. However, to extract phenolic compounds with SFE, co-solvents are often required to increase the solubility of phenolic compounds (especially phenolic acids including gallic acid, methyl gallate, and caffeic acid) in the solvent. Radzali et al. (2020) studied the effect of different co-solvents (ethanol, water, methanol, 50% ethanol–water, and 70% methanol–water) used in SFE by testing the antioxidant capacity of their extracts and found that 70% ethanol–water co-solvent was efficient in increasing the extraction quality of SFE.

Phenolic compounds which are often extremely sensitive to the environment, temperature, oxygen, and light may become the reason for degradation which is not expected. So, compared with conventional extraction methods, SFE is a relatively clean extract that avoids oxidation that causes phenolic compound degradations. (Bleve et al. 2008; Vatai et al. 2009). However, the high cost of SFE is a reason it is not widely applied. Therefore, SFE is mainly used for products with high value (Sunarso and Ismadji 2009).

SFE was proven by Liu et al. (2013) as an effective method that could be used for phenolic compound extraction. Factors that can affect extraction efficiency include temperature, pressure, number of extractions, and extraction time. And for the total phenolic content obtained by SFE is mainly affected by the number of extractions. The best processing method of SFE is considered 2 h at 50 °C with 350 bar pressure for each time of extraction. The extraction quality was also compared by Ashfaq et al. (2021); in their research, they compared the extraction quality of SPE and conventional extraction from tea and use the extracts as coriander sauce preservatives. The total polyphenol content of SPE extracts (224.40 GAE/100 mL) was higher than conventional extraction extracts (208.31 GAE/100 mL), and similar situations also occur in a series of antioxidant assays.

### Pulsed electric field extraction (PEF)

PEF is a non-thermal extraction method that applies short, high voltage pulses to destroy membrane structures and release the content which increases the extraction yield. Several factors may affect the efficiency of PEF including field strength, specific energy input, pulse number, and temperature.

Intact cytomembrane present in plant cells acts as a semipermeable barrier that controls the substances in or out of the cells. Therefore, PEF treatment is a method that could disintegrate the cell membrane and further increase the permeability of the cell wall to allow more bioactive compounds released into the solvents. Due to the structural disintegration of the cytomembrane of the cell, the selective permeability properties will be destroyed which allows more substance to come out during extraction. In addition, this process does not require heating and does not generate much heat which avoids thermolabile compounds’ degradation (Puértolas et al. 2012).

Liu et al. (2018) reported in their study the extraction of phenolic compounds from red onion was conducted at an electric field intensity range from 0.7 to 2.1 kV/cm and treatment times from 30 to 90 pulses under 25 °C. As a result, the extractions of phenolic compounds and flavonoid compounds were increased significantly when the electric field intensity and treatment of time increased. Liu et al. (2018) compared PEF‐assisted water with Soxhlet extraction and found the extraction yield of phenolic compounds and flavonoid compounds of PEF was only 1/3 and 1/2 of Soxhlet extraction, respectively. In addition, PEF also has its disadvantages which include the reversibility of the membrane changes, air bubbles making the process less effective, and the efficiency of the method depending on electric field strength and electrode gap (Joannes et al. 2015).

### Enzyme-assisted extraction (EAE)

Enzymes can hydrolyze the components of the membranes in cells which break their selective permeability properties and release the compounds to increase the extraction rate. The structure of the cell membrane is mainly formed by macromolecules polysaccharides and proteins, and under high-temperature, proteins will denature which will affect the extraction efficiency. Therefore, enzymes like cellulase are applied in extraction as a nonthermal and nontoxic treatment to increase extraction efficiency.

In the old tea leaves extraction experiment done by Hai et al. (2016), they found that compared with samples treated with cellulase, the untreated group has a lower total polyphenol content. And when the concentration of enzyme increased, they found the total polyphenol content also increased and reached a peak of 74.45 mg GAE/g at 2%v/w of enzyme concentration. But when the cellulase concentration raise even further, the total polyphenol level and the polyphenol purity decrease. The reason for this phenomenon may be caused by excessive enzyme-hydrolyzed cell wall polysaccharides which release more impurities that decrease the total polyphenol content and purity (Baye et al. 2015).

However, compared with the chemical- and physical-assisted extraction methods, EAE is more complicated. To obtain extracts of high quality, EAE requires a detailed understanding of the samples including the composition and the enzyme that could be applied for helping extraction. Also, enzyme as a biological protein, its activity is affected by many factors, including pH, temperature, and substrate concentration.

## Separation and characterization

The extracts obtained from the methods mentioned above are complex and contain a variety of natural materials and impurities that require separation to obtain the target chemical. Based on the physical and chemical differences of different compounds, modern technology has enough capabilities to separate them from the extracts.

Generally, to extract phenolic compounds, scientists and industries will choose solvent extraction which produces a mixed product that needs separation to improve its quality. Due to the characteristic of the target compound and the use of the final product, the separation method needs to be carefully selected. Commonly, separation is a purification method for obtaining purified compounds which is often combined with characterization methods to figure out different compounds. In food industries, separation methods can also be used for component quantification to figure out the nutritional list. For phenolic compounds, their biological effects especially the antioxidant effect can be tested by the separated compounds in animal testing. It is also a requirement to separate and purify the extracts, because during testing, the characterization of different compounds may affect each other and lead to a false result.

The basic method of separation is centrifugation which is a way of simulating multiple gravities with a high-speed rotation that can separate impurities from extract in the form of precipitates (Escribano-Bailon 2003). Ultrafiltration is another method that could be applied in phenolic compound separation, but due to the mechanism of ultrafiltration being based on particle size, it also has its limitations when the particle size does not match the filtration membrane size (Ajila et al. 2011). The concentration of extracts is often done in rotary evaporator to purify and separate the specific compound from extracts. And phenolic compounds in the mixture can be separated by solvent separation methods and chromatographic methods.

### Membrane filtration (MF)

In most cases, the supernatant collected after centrifugation is still complex and diluted which needs to be concentrated and separate target phenolic compounds from it. But due to the existence of some heat-sensitive compounds like phlorotannins, scientists and industries cannot always evaporate the extracts. Therefore, membrane filtration (MF) is used as a nonthermal separation method that allows concentration at low temperatures. The principle of MF is using a semipermeable membrane which allows smaller molecules to pass through and block larger molecules. Due to the pore size applied, MF can be classified as microfiltration, ultrafiltration, and nanofiltration. Most phenolic compound studies applied MF as the first rough concentration of extracts (El-Mergawi et al. 2016; Giusti et al. 2019; Saleh et al. 2011) which have been proved to be very efficient phenolic compound classification.

According to the study done by Paraskeva et al. (2007), MF could separate phenolic compounds easily from olive mill wastewater. After further concentration, they obtained a concentrated phenolic product of 28 g/L GAE. But they found the product is unable to be further concentrated due to the high viscosity. Membrane is an easy-applying method but it still has its limitations. According to the studies done by Lu et al. (2011), the affinity for membranes of phenolic compounds and the membrane material characteristics may lead to quantitative and qualitative differences. These differences are affected by the molecular size and the solute structure differences which can control the retention. For example, compounds with larger size and less polar like pectin and protein can occasionally pass through the membrane pore and keep a moderate retention percentage while smaller and more polar compounds like phenolic acids, stilbenes, and sugars may gather at the membrane surface causing a 60% raise of rejection percentage. Yammine et al. (2019) mentioned in their work that to separate phenolic compounds with smaller sizes like monomeric, polymeric proanthocyanidins, and anthocyanins, thinner membrane film could be applied.

### Solid-phase extraction (SPE)-GC/LC (gas chromatography/liquid chromatography)

SPE is a separation method that allows extracts to pass through a solid stationary phase to separate the desired compounds due to their affinity. To separate, concentrate, and clean-up, SPE is often used and compared with other separation methods; the time required for SPE is shorter, and the recovery rate is relatively higher while the SPE is easy to operate; if organized properly, the device even has the possibility of automation. Disposable cartridges are often used in modern SPE devices, but due to the high price of disposable cartridges, the processing is considered low reproducibility, and the flow rate is hard to control during elution. SPE disks are designed as a substitute for cartridges which use less solvent than cartridges when the elution is performed directly by the mobile phase. SPE disks can decrease evaporation to dryness and reconstitution which allows the extraction process more controllable. However, SPE disks are also very expensive even higher than cartridges (Nováková and Vlčková, 2009).

As a pre-treatment of chromatographic analysis, SPE can be directly combined with the analysis devices composed of SPE-LC or SPE-GC. According to the introduction from Xing et al. (2007), the analysis device once optimized with SPE can offer higher speed, lower detection limits, and better reproducibility and separate methods. And Lu et al. (2011) mentioned an improvement of online SPE-LC–MS/MS which uses multiwalled carbon nanotubes as SPE sorbents for the analytes’ online extraction and clean up. Mencin et al. (2021)reported a successful application of SPE with LC–MS; they compared SPE with liquid–liquid extraction and found higher extraction efficiencies of total and individual phenolics and also higher antioxidant activities in SPE method. Similarly, Liberatore et al. (2001) showed that the SPE technique can be successfully used for the extraction and quantitation of phenolic compounds in virgin olive oils, and the extracted compounds could be further characterized after analyzed by GC–MS.

### Liquid chromatography–mass spectrometry (LC–MS/MS)

The principle of LC is based on the affinity differences of compounds which affect the interactions of compounds with the mobile and stationary phases. Then the compounds will be eluted off the column then transformed into gas phase and ionized for mass analysis. By converting the molecules to an ionized state then based on their mass to charge ratio (*m/z*), the mass spectrometers can analyze the ions and any fragment ions that are produced during the ionization process. This is a typical mass spectrometer’s working principle by combining an ionization device with an ion analysis device (Pitt 2009).

For phenolic compound analysis, liquid chromatography with tandem mass spectrometry (LC–MS/MS) have been proven to be effective at low quantification limits. So, we can conclude that LC–MS/MS is one of the most reliable methods for phenolic compound characterization. But due to the precision of the instrument of LC–MS/MS, this method also has its limitations. If the instrument is contaminated, the operator may not be able to find the problem and leading to a false result. And due to the high price of the instrument, LC–MS/MS is not easy to popularize. Because the selection of analytical methods is based on the matrix and the characteristic of phenolic compounds, LC–MS/MS cannot suit all analytical situations. Successful applications of LC–MS/MS were widely mentioned by many researchers, including the characterization of phenolic compounds in custard apple fruit done by Du et al. (2021) who successfully characterized 85 phenolic compounds; Chou et al. (2021) identified a total of 73 compounds, 11, 31, and 49 kinds of unique phenolic compounds in ginger, lemon, and mint respectively by LC-ESI/QTOF-MS; 80 phenolic compounds were identified from chicory and lucerne (Iqbal et al. 2021); A total of 84 phenolic compounds were identified through LC–MS/MS in Australian grown herbs (Ali et al. 2021).

### Gas chromatography–mass spectrometry (GC–MS)

The basic principle of GC*–*MS is similar to LC*–*MS/MS; the main difference is the mobile phase used in the system. Due to the high efficiency and fast separation of gas chromatography (GC), GC is considered an ideal method for phenolic compound separation and characterization. James and Martin (1952) first described this method as a separation method for small carboxylic acids mixture. Different from LC, the mobile phase in this method is a gas stream which is also called carrier gas. This method experienced a long development; packed-columns were first used in this method, but after small carboxylic acids were introduced into GC, the power was improved significantly which was first made by glass; but in 1976, glass was replaced by fused-silica capillary columns which was a great improvement of GC (Niessen 2001).

Zhang and Zuo (2004) applied GC*–*MS in phenolic compound classification in cranberry juice and plasma after consuming cranberry juice. According to their studies, sixteen phenolic compounds were identified in cranberry juice while in plasma samples, only a few benzoic acids and phenolic acids could be found. The advantage of this method includes sensitivity, specificity, and good repeatability which can be also used for phenolic compound antioxidant measurement.

### High-performance liquid chromatography (HPLC)

HPLC is a separation and characterization method that has been widely accepted by many scientists and is a useful tool for phenolic compound analysis other than LC*–*MS and GC*–*MS. Commonly, HPLC is combined with different detectors to analyze phenolic compounds such as ultraviolet–visible (UV) and photodiode array detector (PDA). A large group of phenolic compounds and their derivatives were characterized in previous studies through HPLC and assisted the researchers in identifying the phenolic classes in plants.

HPLC consists of three parts, which include a column which is a stationary phase, a pump that drives the movement of the mobile phase, and a detector that collected data (Martin and Guiochon 2005). The mechanism of retention time in HPLC is similar to LC*–*MS and GC*–*MS which depends on the interaction between mobile and stationary phases (Xiang et al. 2006). Common solvents used in this method are a combination of water and organic liquids like acetonitrile. Varied mobile phase composition during analysis is known as gradient elution which is aimed at separation (Abidi 1991; Xiang et al. 2006). As Abidi (1991) mentioned in his work, the gradient that separates the compounds is also based on the difference in affinity with the mobile phase of different compounds. Therefore, due to the characteristic of the stationary phase and the analyte, the solvents and gradient dependencies could be decided. Research on mangiferin quantification was done by Imran et al. (2016), and in this study, HPLC was successfully used and obtained the variety of mango with the highest mangiferin content among the five tested varieties of mangoes.

### Capillary electrophoresis (CE)

CE compared with HPLC is a relatively new separation method that has the potential to become an alternative to HPLC and become a wider used method for phenolic compound characterization methods. As Skoog et al. (2017) mentioned in their work, CE is an analytical separation method that uses an electric field to separate the components of a mixture. It is electrophoresis in a capillary, a narrow tube. Hence, the components of the mixture are separated based on their electrophoretic mobility. Only the ions are affected by the electric field while the neutral species remain unaffected. The rate of a molecule that moves through the capillary depends on the strength of the electric field. The analytes separate as they migrate due to their electrophoretic mobility, and are detected near the outlet end of the capillary where a detector is often set. The output of the detector is sent to a data output and handling device such as an integrator or computer. The data is then displayed as an electropherogram, which reports detector response as a function of time. Separated chemical compounds appear as peaks with different migration times in an electropherogram.

As Tsao and Deng (2004) mentioned in their studies, CE is a separation method with more advantages than HPLC, which include the sample size required for CE being smaller than HPLC, non-parabolic fronting bringing CE higher efficiency, the time required for analyzing is shorter, low cost, and low or no requirement of organic solvent.

### Countercurrent chromatography (CCC)

Countercurrent chromatography is a separation method that moves two liquid phases with respect to one another to form a liquid–liquid chromatographic system not requiring a solid support (Ito and Bowman 1970). This method was first introduced by Ito and Bowman (1970) in the late 1970s and has been widely used for phenolic compound separation and purification. The mechanism of CCC is based on the compound distribution between two immiscible liquids which are called mobile phase and stationary phase, respectively. The previous report mentioned that CCC does not have several band-broadening mechanisms caused by the action of random imperfection in the flow stream like conventional chromatography because CCC does not have a solid support matrix in the column (Wang et al. 2015). Therefore, CCC has a less complex mathematical description of the separation process than conventional liquid–liquid chromatography because in the conventional case, the liquid stationary phase is retained on a solid support.

CCC has been used for the separation of phenolic compounds from plants. He et al. (2009) reported a successful application of high-speed CCC for the preparative isolation of the minor phenolic compounds from the ethyl acetate extracts of black currant fruit. In their study, high-speed CCC separation was performed with a two-phase solvent system composed. As a result, 0.8 mg of protocatechuic acid, 1.0 mg of caffeic acid, 0.5 mg of 4-hydroxybenzoic acid, and 2.5 mg of myricetin were separated from a total of 500 mg crude sample by a one-step high-speed CCC operation.

### Centrifugal partition chromatography (CPC)

Centrifugal partition chromatography is a developed method of CCC which was introduced by Murayama et al. (1982). CPC is a type of hydrostatic countercurrent chromatography and like other same types of methods which are based on the phenomenon of liquid–liquid partitioning between two immiscible liquid phases that stay at equilibrium. But in the case of CPC, the mechanism of stationary phase retention is significantly different from others; the centrifugal field in the rotor in a one-axis centrifuge generates hydrostatic force to conduct stationary phase retention (Bojczuk et al. 2017). However, the efficiency of the separation in CPC is affected by a variety of parameters which include the sample properties in the solvent system, physical properties of the solvent system, parameters of the instrument, and the method. CPC does not need an expensive solid stationary phase, and compared with conventional CCC separation methods, both the quantity and quality requirements regarding the solvents/phases to be used could be much more similar in terms of their properties (Santos et al. 2018).

In previous studies, in order to purify caffeic, ferulic, and protocatechuic acids, CPC was applied and achieved an efficient separation of the three phenolic acids (Santos et al. 2018). Alherech et al. (2021) also reported in their study that CPC is an effective technology that could provide scalable access to valuable chemicals from lignin and other biomass-derived feedstocks.

## Phenolic content and antioxidant activity determination methods

To test the phenolic contents and the antioxidant capacity of plant samples, a series of antioxidant assays were applied. The most concerning values are the phenolic content in plants. Therefore, the phenolic, flavonoids, and condensed tannins content were often tested in many studies. And to test the antioxidant capacity of plant extracts, assays including DPPH test are also useful tools in many pieces of researches.

### Total phenolic content (TPC)

To evaluate the health value of plants, the TPC value can be an important indicator (Khomdram and Singh 2011). Factors including extract volume, reagent selection, reaction time, color development temperature, measurement wavelength, and reference substance may affect the TPC values (Abdelkader et al. 2014).

**Fig. 1:**
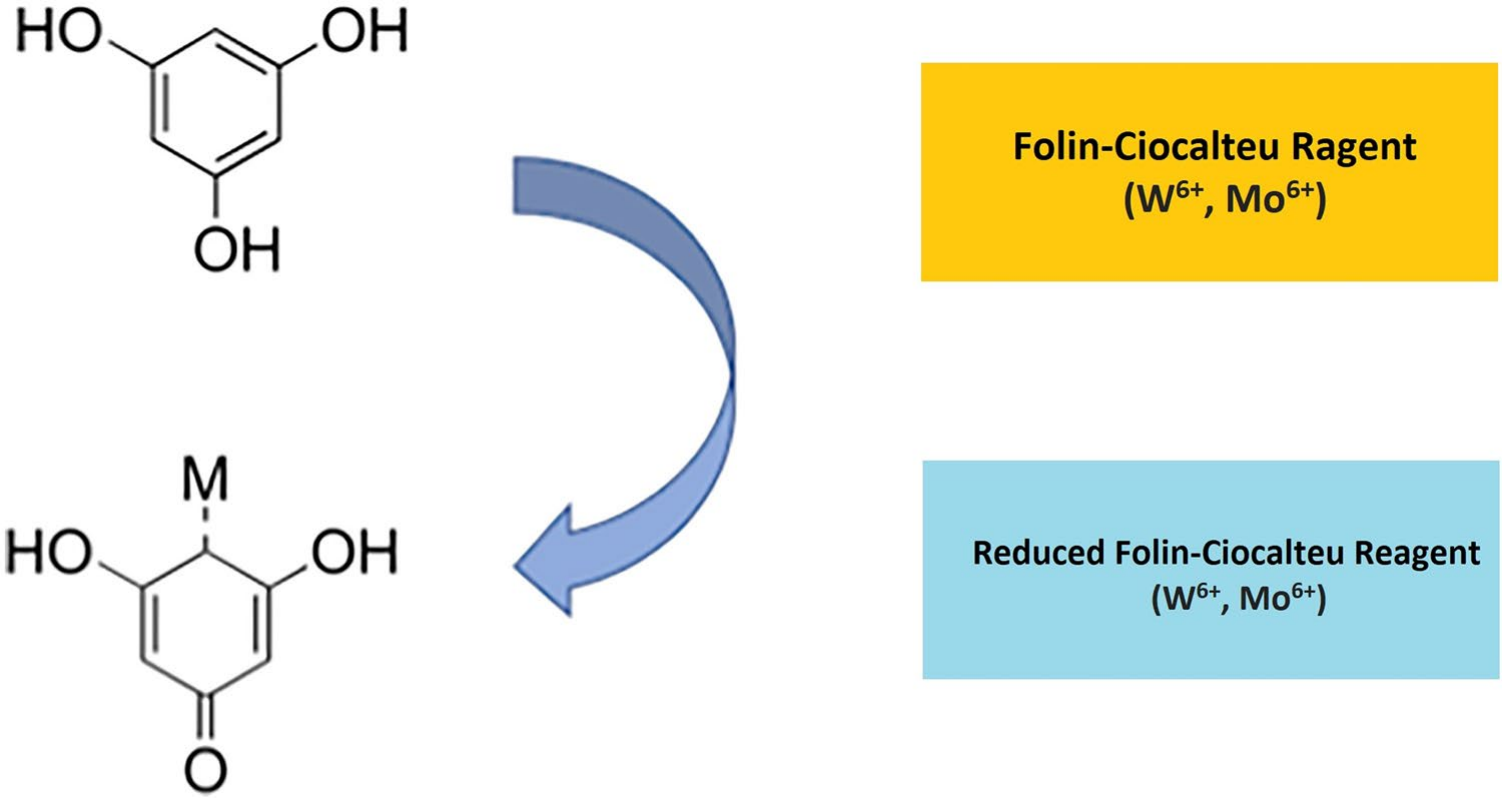
Principle of Folin–Ciocalteu method (Ford et al. 2019)

The principle of measuring the TPC in plant materials is based on the color change reaction between polyphenols and Folin-Ciocalteu reagent (FCR) which can form a blue complex then can be quantified by instruments like spectrophotometric microplate reader (Abdelkader et al. 2014; Agbor et al. 2014). As Agbor et al. (2014) mentioned in their study, the blue complex is a phosphotungstic-phosphomolybdenum complex. And due to the alkaline solution and the concentration of phenolic compounds, absorbance of this matrix will be different which makes the quantification of phenolic compound concentration available (Blainski et al. 2013). FCR is a very sensitive and accurate method which has been proved by several scientists that it can provide accurate and specific data for TPC measurement as color responses were common and intuitive in their research (Fig. 1) (Agbor et al. 2014).

### Total flavonoids content (TFC)

**Fig. 2:**
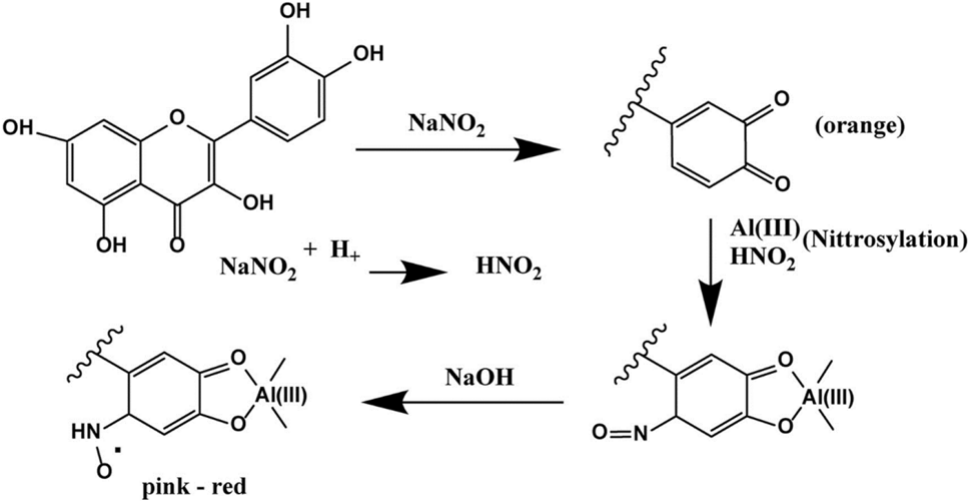
Formation of flavonoid complex with AlCl_3_ (Shraim et al. 2021)

The flavonoid content in plant materials is also an important parameter in evaluating plants’ nutritional value. So, to test the TFC of plant materials, Al-flavonoid complexation method is often applied. In 1960, the original method was designed by Christ and Müller (1960) for herbal materials-analyzing purposes, and after many years of modification, it becomes the modern form. There are two main procedures for applying Al-flavonoid complexation reaction. Commonly, a concentration of 2–10% (m/v) AlCl_3_ solution is added to a sample then measure the absorbance at 404–430 nm after 2–60 min, and different flavonols like quercetin or galangin and flavan-3-ol catechin can be used as equivalent standard. The other procedure was a *o*-diphenol determination method used by Barnum (1977); the method is based on the color change of aromatic ring bearing a catechol group with Al (III) which is an orange complex and will turn pink-red when added NaOH. Then the absorbance can be measured with instruments like a spectrophotometric microplate reader at 510 nm (Fig. 2).

### Total tannins content (TTC)

To test the TTC in pant materials, the vanillin method is often applied due to its color complex formation mechanism. Factors that affect this reaction include solvent and reagent selection, concentration, reaction time, temperature, and the reference standard selection (Dalzell and Kerven 1998; Makkar and Becker 1993; Scalbert 1992; Sun et al. 1998).

**Fig. 3:**
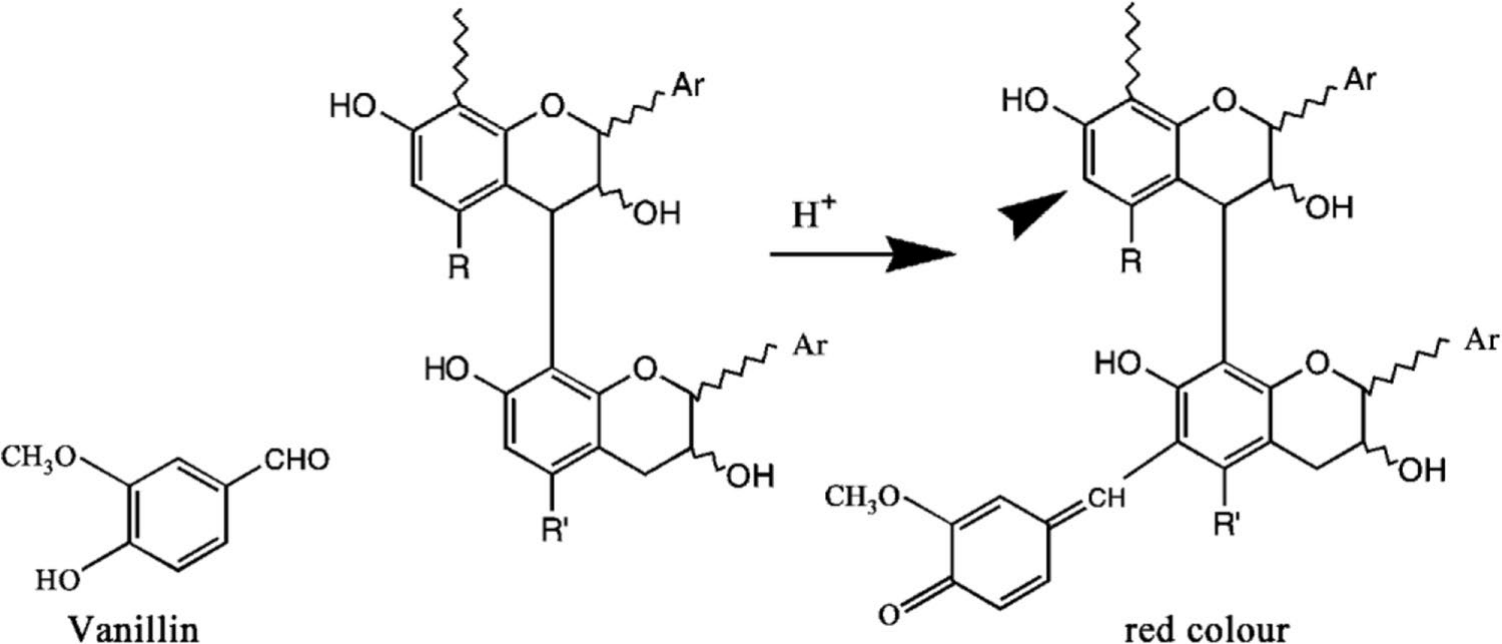
The mechanism of vanillin assay for condensed tannins (Schofield et al. 2001)

In the vanillin assay method, sulfuric acid is the most often used catalyst but if the normality of acid is too high, the absorbance at 500 nm (A500) will also increase (Scalbert 1992; Sun et al. 1998). As Bae et al. (1993) and Sun et al. (1998) discussed in their work, the rise of water content can dramatically decrease the A500 in this test. Waterman and Mole (1994) and Sun et al. (1998) both mentioned the importance of temperature control in their work, and they also found an 11% increase of A500 can be obtained for each 1.4 °C increase in their research. However, the result of the research done by Sun et al. (1998) was not so severe, but they also mentioned that to achieve the highest absorbance, the TTC test should be conducted under 25–30 °C (Fig. 3).

### The DPPH (2,2-di(4-tert-octylphenyl)-1-picrylhydrazyl) test

The DPPH test is a free radical scavenging activity testing method. The mechanism of reaction with antioxidants is a color change from a deep purple (DPPH•) to pale yellow (DPPH) after reaction with peroxyl radicals ROO^•^ (Benzie and Strain 1999). DPPH^•^ presents as a monomer in both solid and liquid states which can solve in organic solvents like methanol and ethanol to react with free radicals which are not soluble in water. As Staško et al. (2007) mentioned in their work, water content should be controlled below 60% to remain good solubility of radicals.

**Fig. 4:**
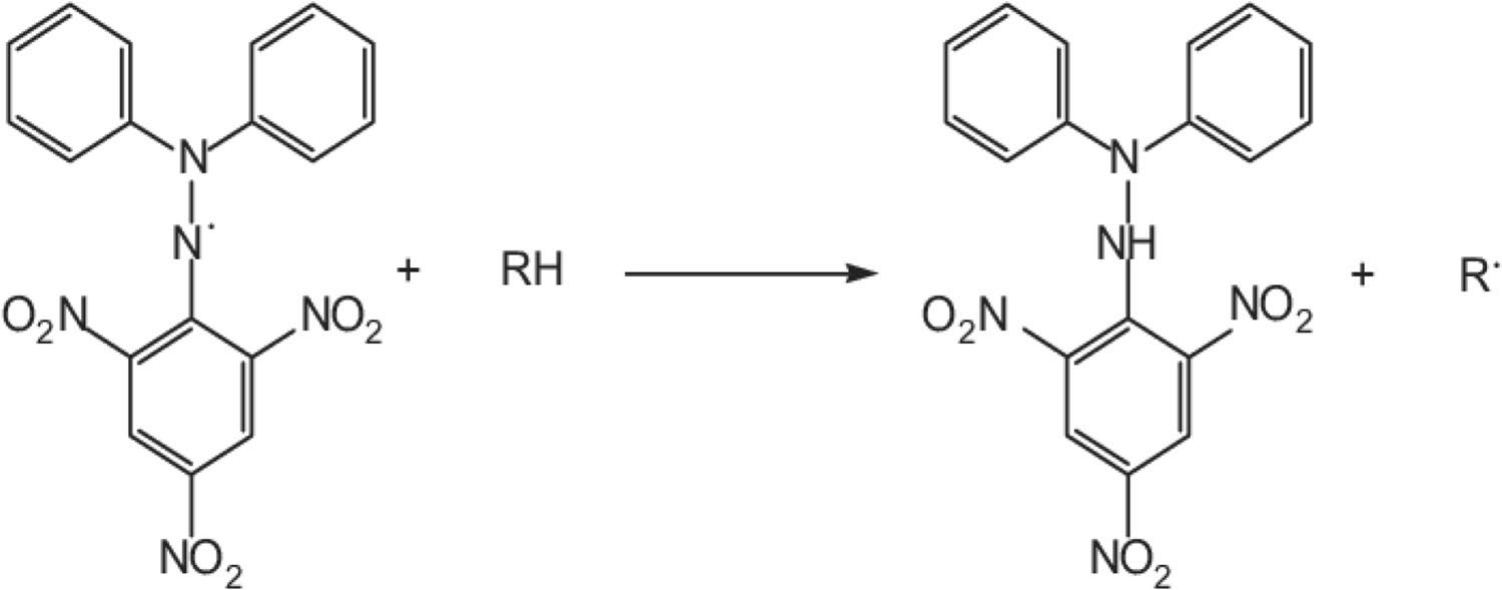
DPPH scavenging mechanisms (Dureja and Dhiman 2012)

The DPPH radical is neutralized after accepting electrons from antioxidants, and due to the color change during this process, the antioxidant capacity can be quantified at 517 nm. There are two main ways of reporting the antioxidant activity measured by DPPH neutralization method which are EC_50_ (the concentration required for the antioxidant to reduce the initial DPPH concentration by 50%) and T_EC50_ (the time required for achieving the equilibrium state with EC_50_) (Foti 2015) (Fig. 4).

### Ferric reducing/antioxidant power (FRAP) test

FRAP test is an antioxidant capacity measuring method based on single electron transfer (SET) method to measure the reduction of ferric ions (Fe^3+^)-ligand complex to the intensely blue ferrous complex (Fe^2+^) (Antolovich et al. 2002). The absorbance is measured at 593 nm, and the antioxidant is positively related to the micromolar equivalents of Fe^2+^ in this test. But different from other SET-based methods, FRAP test requires a low pH environment to maintain iron solubility which decreases the ionization potential and increases the redox potential (Hagerman et al. 1998).

**Fig. 5:**
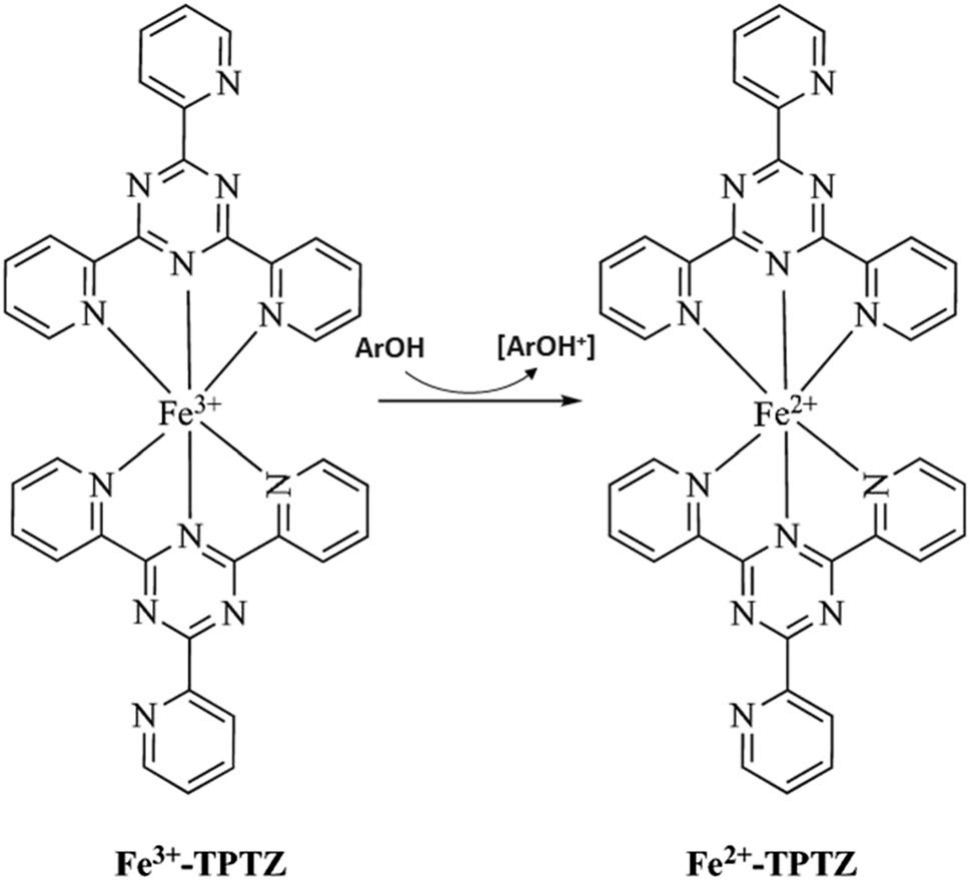
The mechanism of ferric reducing antioxidant power (FRAP) reaction (Munteanu and Apetrei 2021)

Tripyridyltriazine (TPTZ) was first used in FRAP test as an iron ion linking ligand, and the substitutes are also used for the same effect (Molina-Dıaz et al. 1998). For example, ferrozine is used for evaluating ascorbic acid’s antioxidant capacity. Potassium ferricyanide is the most common ferric reagent used in modern FRAP tests. Based on spectrophotometry, the reducing power of the tested material could be quantified after the blue color was generated. Generally, there are two principles of blue color formation that do not affect the result. The first one is after Fe^3+^ is reduced to Fe^2+^, it will bind with ferricyanide forming blue complexes. The second one is ferrocyanide reduced from ferricyanide can bind with Fe^3+^ to form the complexes (Fig. 5).

### Ferrous ion chelating ability (FICA) assay

FICA assay is a test for antioxidant capacities and its principle is based on the transition metal ion chelating ability like Fe^2+^ and Cu^2+^. Ferrous chloride (FeCl_2_) is often used in this kind of determination test to provide Fe^2+^. The typical procedure of FICA test is first mixing the sample with FeCl_2_. Then the mixture after incubation will be added ferrozine to form color complexes in dark environment incubation. The absorbance will be measured at 562 nm. To quantify the chelating ability, ethylene diamine tetraacetate (EDTA) is used as a positive control.

**Fig. 6:**
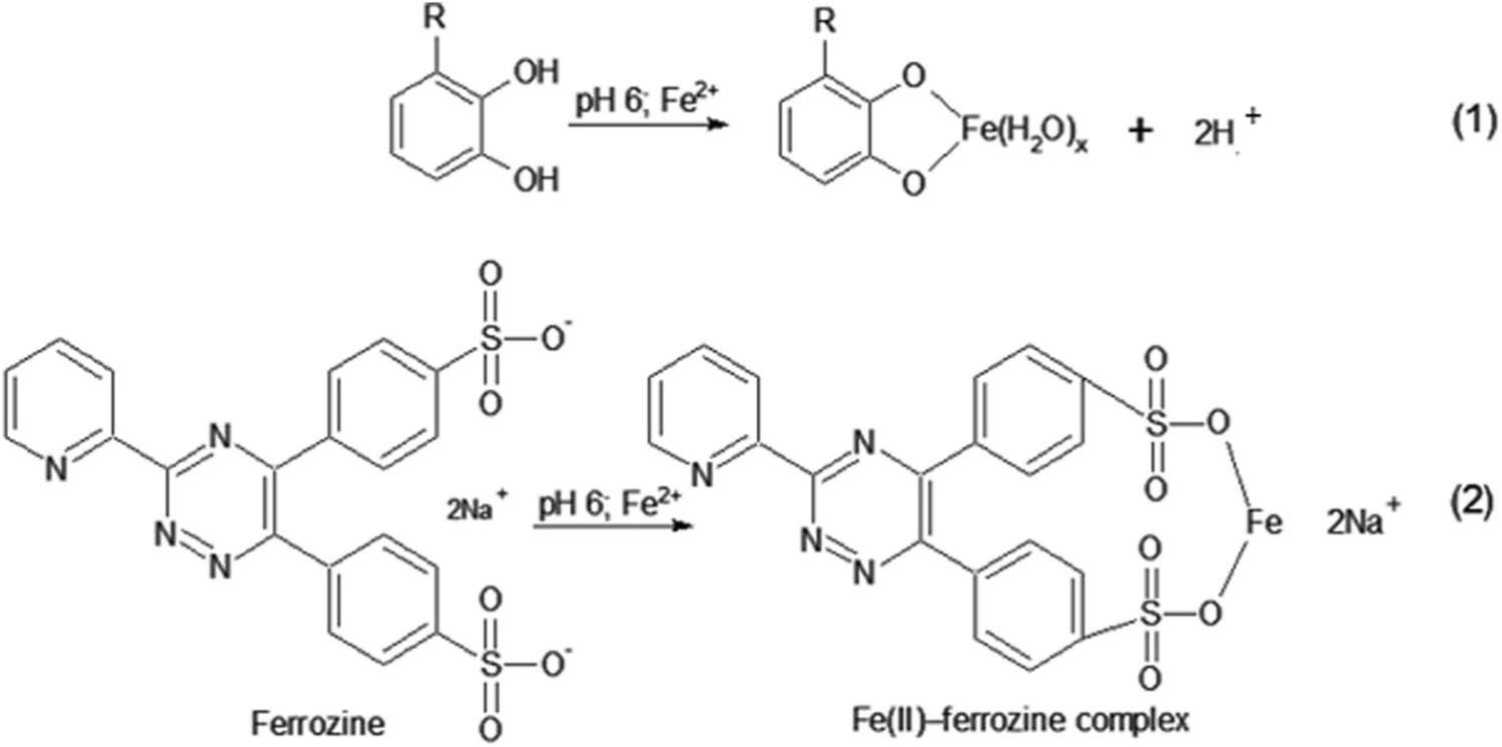
The principle of ferrous ion chelating ability (FICA) assay (Santos et al. 2017)

Md Yusof et al. (2013) applied this test to measure the chelating ability of *Pandanus pygmaeus*. In their test, only the methanol extract of *Pandanus pygmaeu* and the EDTA group showed chelating ability. When they increased the sample concentration in extracts, the absorbance of ferrous and ferrozine complex decreased, which indicates the positive relation between chelating ability and sample concentration. As they reported, the peak of chelating activity is obtained at 10,000 µg/mL while the chelating percentage is at 85.39%. The chelating ability is expressed as an IC_50_ value in this test; lower IC_50_ value means the chelating activity is higher, and in the research done by Md Yusof et al. (2013), they found EDTA has a lower IC_50_ than the tested extract which means compared with EDTA, *Pandanus pygmaeu* extract is a moderated metal-chelating agent (Fig. 6).

### The 2,2’-azino-bis-(3-ethylbenzothiazoline-6-sulfonic acid) (ABTS) radical scavenging assay

The ABTS radical scavenging assay is another method used for testing the antioxidant capacity of plant extract. In 1993, ABTS test was first applied by Miller et al. (1993) based on the principle of the interaction between antioxidants and ABTS^+^ which indicates the ability to scavenge free radicals. Due to a bluish-green color complex formed during this test, the activity can be quantified by measuring the absorbance at 734 nm, whose intensity decreases the strength of antioxidant capacity. Because of the presence of powerful antioxidant agents, ABTS can be transformed into ABTS^+^ which is the main factor that affects the degree of discoloration of the blue–green color, which will be reflected as absorbance decreases at 734 nm.

**Fig. 7:**
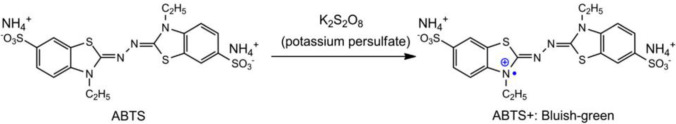
The mechanism of ABTS radical scavenging activity assay (Xiao et al. 2020)

ABTS tests have been widely used for antioxidant capacity evaluation and for *p*-hydroxybenzoic acids and polyphenolic compounds (Solís-Oba et al. 2005). As Arnao et al. (1990) mentioned in their work, this method can also be used for some varieties of flavonoids by using peroxidase. However, both methods are based on the ABTS–polyphenol complex formation under enzymatic reaction. ABTS radical scavenging assay does not have a strict pH requirement which allows a wide range of evaluation and is beneficial for studying the pH effect on food components’ antioxidant mechanisms. In addition, ABTS radical is both water and organic soluble which make it able to evaluate the antioxidant capacity of oil and water-soluble components (Fig. 7).

### Hydroxyl radical scavenging activity assay (^•^OH-RSA)

^•^OH-RSA assay is an antioxidant testing method based on the Fenton reaction (Fe^3+^-ascorbate-EDTA-H_2_O_2_ system), which measures the hydroxyl radicals generated from hydrogen peroxide. In the presence of iron, hydrogen peroxide can process a set of reactions called the Fenton reaction which can generate hydroxyl radicals then scavenged by antioxidants. The quantification in ^•^OH-RSA assay is conducted on malonaldehyde produced during the 2-deoxyribose degradation then condensed with thiobarbituric acid forming a yellow complex. This complex absorbance will be measured at 510 nm.

**Fig. 8:**
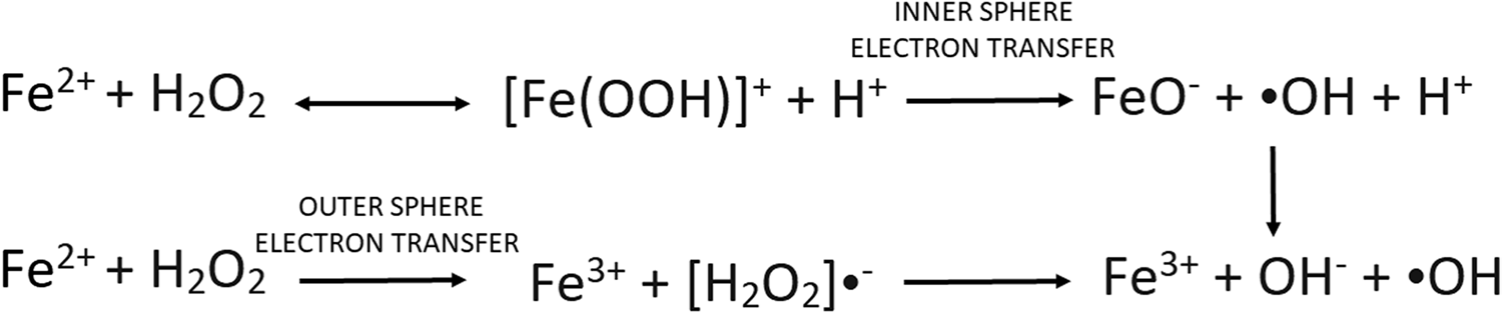
Fenton reaction mechanism (Salgado et al. 2013)

Testing the hydroxyl radical scavenging activity is meaningful, especially in the medical area. Free radicals can form under the Fe^2+^ existing environment which can be even driven by non-reactive radicals. Therefore, chelating Fe^2+^ can be an effective treatment of oxidative stress diseases. However, the toxicity of chelators also needs to be considered. Chelators may be toxic which requires a balance between the toxic damage and the positive effects of antioxidize effect. Franco et al. (2019) had already introduced the achievement of using antioxidants to protect red blood cells from oxidative damage which is based on the mechanism of chelators can form stable complexes with free radicals then excreted by feces and urine. But due to the serious side effect of most chelating agents, discovering potential chelators from natural ingredients is very valuable for scientists and may be less harmful to patients. Therefore, ^•^OH-RSA assay for testing the hydroxyl radical scavenging activity of plant extracts is important which may help discover new free radical chelators (Fig. 8).

### Reducing power assay (RPA)

RPA is a more general test of the antioxidant which was first described by Oyaizu (1986). The principle of the RPA test is based on the reduction ability of the phenolic compounds that could reduce the Fe^3 +^/ferricyanide complex to the ferrous form which changes from yellow to a green–blue color. Then the reducing power can be determined at the absorbance of 700 nm. This test needs to take special care with the heating process, with the test tubes partially closed, so that there is no solvent concentration process, and so it can be validated and determine the real concentration.

The RPA test was used by Martorana et al. (2013) based on the RPA method mentioned by Oyaizu (1986) to test the reducing power of pistachio extracts. They used methanol/water (2/1) solutions to extract phenolic compounds from pistachio then the extract was mixed with sodium phosphate buffer and K_3_Fe(CN)_6_ before an incubation under 50 °C. After incubation, trichloroacetic acid was added to the sample then centrifuged, and the supernatant was collected then added distilled water and ferric chloride solution. As a result, the formation of blue-green complex can be used for quantifying the reducing power of pistachio extracts at an absorbance of 700 nm. Ascorbic acid was used as an equivalent standard in their test, and the reducing power of the pistachio extracts was successfully tested as 1.13 mmoles AAE/g (seed) and 2.69 mmoles AAE/g (skin).

### Total antioxidant capacity (TAC) test

TAC test is a general test of the antioxidant capacity of plant extracts which Jayaprakasha et al. (2002) mentioned in their research as a phosphomolybdate method-based test using α-tocopherol as an equivalent standard. The test is performed by mixing the extracts with a special reagent which is a mixture of sulfuric acid, sodium phosphate, and ammonium molybdate. The mixture will then be incubated at 95 °C for 90 min, and the absorbance is measured at 695 nm after cooling. α-tocopherol will be also tested with the same procedures as a standard. As Jayaprakasha et al. (2002) reported, α-tocopherol can make a standard with good performance under the same conditions and was selected as the standard. Therefore, the TAC value can be expressed as µg equivalents of α-tocopherol.

## Summary

As important raw materials, plants play a significant role in the medical and food area. Plants supplement human’s nutritional needs in their diet and can be widely used in the treatment of various diseases. The high content of phenolic compounds in some plants even raised a great interest from many scientists. The potential of applying the high antioxidant capacity of phenolic from plants might be valuable. Therefore, developing methods that could be used for phenolic compound extraction, separation, and characterization are meaningful to evaluate the nutritional value of these compounds. For extraction methods, the most commonly used method is still conventional maceration extraction which had been applied in most of the research in this area. However, to improve the efficiency of solvent extraction, techniques including ultrasonic, Soxhlet extraction, and enzymes are also applied. But due to the limitation of some methods, it is important to select the methods carefully to maximize the extraction efficiency of target components.

Considering the analytical methods used for phenolic compounds determination including HPLC, LC–MS, and GC–MS they all appeared to first separate the compounds from the extract and then analyze the characteristics of the compounds to classify them. Improvements in the characterization method of evaluating the chemical structures of these compounds have been developed over several decades, and the database of most phenolic compounds has been clarified which is beneficial for analyzing the phenolic in plants. Similarly, a series of assays have been developed for testing the content and the antioxidant capacity of phenolic compounds in plants. These methods are all considered to be reliable tools in chemical analysis of plants. However, although all of these methods are proven to be reliable in many plant extracts, some of them lack data on application to a wider variety of plants which is worth studying in the future. Current research of the phenolic compounds in plants and methods used for characterizing the phenolic types and testing their antioxidant capacities are considered to be a great starting point. But due to some of the methods having their limitations, it still requires further research to test the reliability of these methods applied to plant extracts.

## Data Availability

Not applicable.
